# Combining citation and productivity metrics through harmonic mean enhances researcher ranking accuracy

**DOI:** 10.1038/s41598-025-30432-4

**Published:** 2025-11-29

**Authors:** Ghulam Mustafa, Muhammad Saeed Khattak, Muhammad Tanvir Afzal, Adnan Akhunzada, Mario Konecki, Nikola Ivković

**Affiliations:** 1 Department of Computing, Shifa Tameer-e-Millat University, Islamabad, 44000 Pakistan; 2 College of Computing and IT, Department of Data & Cybersecurity, University of Doha for Science and Technology, Doha, 24449 Qatar; 3 Faculty of Organization and Informatics, University of Zagreb, Pavlinska 2, 42000 Varaždin Croatia

**Keywords:** Author assessment parameters, Parameter ranking, Multi-layer perceptron (MLP), h-Type indicators, Neuroscience domain datasets, Computational biology and bioinformatics, Mathematics and computing, Neuroscience

## Abstract

Addressing the challenge of predicting scientific impact and ranking researchers is a complex yet critical task, drawing significant attention from scholars across diverse fields. This effort plays a key role in improving research productivity, supporting decision-making processes, and advancing methodologies for scientific evaluation. Over time, various metrics such as citation counts, total publications, hybrid methods, the h index, and h-type indicators have been introduced to identify influential researchers. Despite these efforts, no single metric has been universally accepted as the best approach, as different metrics serve varying purposes and contexts. This study presents a novel index developed through comprehensive analysis of a dataset comprising 1060 Neuroscience researchers, including both awardees and non-awardees. The initial phase of the research involved evaluating specific metrics to determine their ability to place awardees among the top 100 researchers, leading to the identification of the five parameters most frequently associated with awardee inclusion. Advanced deep learning techniques were then applied to refine the selection, pinpointing the top five influential parameters and assessing the disjointness in their outputs. To further enhance the findings, seven statistical models were examined for their ability to combine the most disjoint parameter pair while retaining their individual strengths. Selecting the most disjoint pair ensures that the ranking process integrates diverse evaluation criteria rather than relying on redundant or highly correlated parameters. This approach captures a broader spectrum of researcher impact, reducing bias and increasing the robustness of the final ranking index. Among these models, the h2 upper and k indices exhibited the highest disjointness ratio at 0.97. Additionally, the Harmonic Mean approach demonstrated superior performance, achieving an average impact score of 0.76, and excelled at preserving the unique features of the selected parameter pair. Based on these results, a new index was formulated using the Harmonic Mean (HM) of the most disjoint pair. This index showed significantly improved performance compared to existing metrics, offering a robust solution for ranking researchers effectively.

## Introduction

Ranking and identifying influential researchers within the scientific community is a critical endeavor with far reaching implications. It not only recognizes individuals deserving of tenure but also facilitates access to research funding, project opportunities, and acknowledgment for significant contributions^1–4^. Additionally, rankings are vital for evaluating academic productivity and achievements. Raheel et al. highlight the importance of a fair and comprehensive ranking system that incorporates educational qualifications^5^. Such a system supports conference organizers by providing an objective reference for selecting keynote speakers, particularly in interdisciplinary or emerging fields where organizers may not always have direct expertise in every specialized domain. Moreover, this also aids students in making informed decisions when choosing research supervisors, guided by the professors’ academic reputation and expertise. These factors emphasize the pressing need for a robust and accurate index to effectively and equitably rank researchers within the scientific community.

Over the past two decades, numerous author assessment parameters have been proposed, with over 70 metrics extensively explored in academic literature^6,7^. These parameters employ a mix of quantitative and qualitative approaches, with certain indices explicitly designed as hybrid models. Early evaluations often relied on straightforward metrics like publication counts^8^ and citation numbers^9^. However, such basic metrics frequently fall short in capturing a researcher’s actual influence. For example, a high number of publications does not necessarily indicate quality, as some authors focus on quantity by publishing in lower impact journals^10^. Similarly, citation counts can be distorted by practices such as self-citations or the strategic use of survey papers to attract citations^11^.

To address these issues, the h index was developed as a metric that combines productivity and impact^12^. Productivity, often measured by publication counts, makes the h index a size dependent metric. Impact, on the other hand, can be categorized into total impact, the total number of citations, which has both size dependent and size independent attributes as a composite measure and specific impact, which refers to the ratio of citations to papers, making it size independent. Although the h index marked an improvement, it has its own limitations, such as disregarding the total citations of highly impactful works and failing to account for the significance of collaborative research^13^. Despite the introduction of numerous ranking metrics, there is still no consensus on a universally accepted standard for researcher evaluation, leaving the subject open to further exploration. Comparative studies across disciplines have demonstrated that the effectiveness of metrics varies depending on the dataset, with no single parameter consistently outperforming others^4,11,14–16^. However, many of these studies focus on a limited set of metrics, restricting their findings and leaving gaps in understanding the broader implications of these measures.

A comprehensive analysis of existing literature reveals several key challenges in the evaluation and application of research indices. One major issue is that many newly developed indices are often tested in theoretical or simulated environments rather than validated using large, domain-specific datasets^17–22^. This limitation reduces their applicability and reliability in practical scenarios. Another challenge is the absence of benchmarking against standardized metrics or established gold standards, which makes it difficult to accurately assess their effectiveness. Although most indices are based on commonly used parameters such as publication counts, citation metrics, and co-authorships, their performance can differ significantly due to variations in their underlying formulas. This inconsistency highlights the need for systematic evaluations of how effectively each index identifies awardees. Furthermore, there is a notable lack of research examining indices based on their ability to identify awardees in a disjointed manner. For instance, a disjointness value of 100 between two parameters implies that one parameter identifies the maximum number of unique awardees with no overlap with the other, resulting in an empty intersection between the awardee sets. Exploring such behavior is essential for refining research indices and enhancing their applicability across diverse academic domains.

This research adopts a comprehensive approach to address the challenges highlighted in previous studies, making several significant contributions. Firstly, it conducts a detailed analysis of author assessment parameters, utilizing a robust dataset from the neuroscience domain that has been referenced in multiple earlier works^4,23^. Secondly, the study examines 64 existing parameters, representing an extensive and unique selection in this field. To further enhance insights, the concept of disjointness is introduced to measure the degree of independence between different bibliometric parameters. Disjointness evaluates how distinct two indices are in identifying top-performing authors, with higher values indicating less overlap and therefore greater independence between the ranked sets. This ensures that parameters combined in subsequent analysis contribute complementary rather than redundant information.

To further enhance analytical insights, the disjointness ratio is calculated for all possible pairs of top-performing indices, assessing their capability to identify distinct awardees without overlap. A comprehensive evaluation of these indices is then conducted using a gold standard dataset to determine the most effective pair, which is subsequently integrated through various statistical models to maximize performance. The outcome of this process highlights the need for a unified metric that not only preserves the strengths of existing indices but also minimizes redundancy and enhances fairness in evaluation. Motivated by these findings and the limitations discussed earlier in the introduction, this study introduces the HK Index-a balanced and harmonically integrated metric that combines the h2 upper index and the k index. This new formulation effectively captures both the intensity of research impact and the consistency of scholarly output, resulting in a more stable and equitable assessment of academic performance across diverse disciplines. The HK Index is particularly suited for comprehensive evaluation contexts such as academic awards, tenure decisions, and inter-field comparisons, where fairness and cross-domain comparability are critical.

This paper is organized as follows: The “Literature review” section summarizes key studies related to the field. The “Methodology” section details the process for calculating and evaluating the indices, as well as the steps involved in creating a new index. The “Results” section highlights the main findings, while the “Conclusion” section provides closing insights and observations.

## Literature

Evaluating the scientific productivity of researchers is essential for various academic and professional decisions, such as selecting award candidates, aligning researchers with suitable projects, advancing careers, granting promotions and tenure, and awarding expert contracts^24^. Over the past two decades, more than 70 metrics have been developed to assess and rank researchers based on their contributions^6^. Initially, assessments primarily relied on the total number of publications^10^. However, this metric often fails to accurately reflect the quality or impact of a researcher’s work, as some individuals may increase their publication count by targeting low-impact journals simply to bolster their numbers. While specific impact is both quality and size dependent, total impact results from the product of publication count (a quantity-based factor) and quality. To address this issue, citation counts were introduced as an alternative metric^9^. While offering a new perspective, this approach also has limitations. For example, newly published papers may take time to accumulate citations, which can disadvantage early career researchers whose significant contributions have not yet had sufficient exposure. Furthermore, citation counts do not always indicate quality, as papers may be cited for critique rather than endorsement, and self-citations can distort citation totals.

To overcome these limitations, Hirsch proposed the h index in 2005^12^, which soon became a popular metric for evaluating research output. Despite its widespread use, the h index has notable shortcomings. For example, it tends to advantage senior researchers by prioritizing long-term productivity and exhibits significant variation across academic disciplines. Moreover, the h index is not particularly sensitive to increased citations for an author’s most impactful work, which limits its ability to represent the true influence of highly cited papers. In response to these challenges, a variety of alternative metrics and extensions to the h index have been developed, such as the g index^17^, t index^18^, ar index^19^, e index^20^, p index^21^, a index^22^, m quotient^25^, contemporary h index^25^, f index^26^, wu index^27^, and q2 index^10^, among others. However, many of these metrics were initially evaluated in theoretical or controlled environments, which limits their relevance and applicability in real-world contexts^28^.

Several studies have evaluated research indices using real-world datasets. For instance, Kolmulski et al.^29^ explored the relationship between the h index and the h2 index, using data from 19 chemistry professors at a university in Poland, and found a strong correlation between the two metrics. Vaan Raan^30^ compared the h index and its variations with data from 147 chemistry research groups in the Netherlands, focusing on group performance rather than individual contributions, and utilized a three-year citation window instead of lifetime citations. Furthermore, Jin et al.^31^ introduced an integrated approach combining multiple indices, such as the h index, r index, and ar index, demonstrating that this combined method provided a more comprehensive evaluation of research impact.

Mane et al.^32^ evaluated the g index and compared it with other metrics, such as the h index, a index, and r index, using data from physicists at Chemnitz University of Technology. Their findings suggested that the g index was a more reliable measure of overall research impact. Xiao et al.^33^ examined 29 variations of the h index and explored their correlations with both the h index and wu index. They discovered that indices with a high correlation to the h index showed weaker correlation with the wu index, implying only marginal improvements over existing metrics. The w index, proposed by Wu^34^, was developed to address the limitations of single-number metrics like the h index and Nq-most, where Nq-most represents the sum average of citations to the q most cited publications (e.g., q = 3), as described in Wu’s work. It enhances the evaluation by considering the significance of key publications, adjusting citations according to the researcher’s authorship position, thus providing a more detailed understanding of impact. While the study noted challenges with self-citations, their impact was found to be minimal.

Ayaz et al.^35^ evaluated the complete-h index using a dataset of awardees from mathematical societies, concluding that it was more effective than other indices. In a similar vein, Raheel et al.^5^ examined publication age and citation-based variants within the civil engineering domain, finding that the wu index performed better than other metrics. Ain et al.^35^ conducted a study in mathematics, demonstrating that fraction count per paper was a more reliable parameter than others. Ameer et al.^36^ found that the r index and hg index were particularly effective for ranking awardees in neuroscience. Additionally, Ain et al.^16^ assessed the correlation between various h-type indicators and award winning researchers in mathematics, identifying the most effective ones. Salman et al.^37^ evaluated indices in the context of multi authorship in civil engineering, concluding that the gf-index outperformed other metrics. However, many studies face the limitation of associating the h index or h-type indicators with awardees who may not have been influenced by these indices due to the timing of their awards. To address this, Usman et al.^38^ selected both awardees and non-awardees from the same post-2005 period for a more accurate evaluation of effective parameters. Alshdadi et al.^39^ used deep learning models to propose rules for identifying influential researchers, achieving an accuracy rate of up to 70% across various domains.

A review of existing literature shows that initial evaluations mainly focused on basic publication and citation counts, which were later expanded to include variations of the h index. However, these methods still fell short of addressing the limitations of current metrics. To overcome this, a thorough study is required-one that examines a wide array of indices using consistent, domain-specific datasets, ranks these parameters using machine learning techniques, and creates a new index that more accurately identifies top-performing researchers than existing methods.

## Methodology

**Fig. 1:**
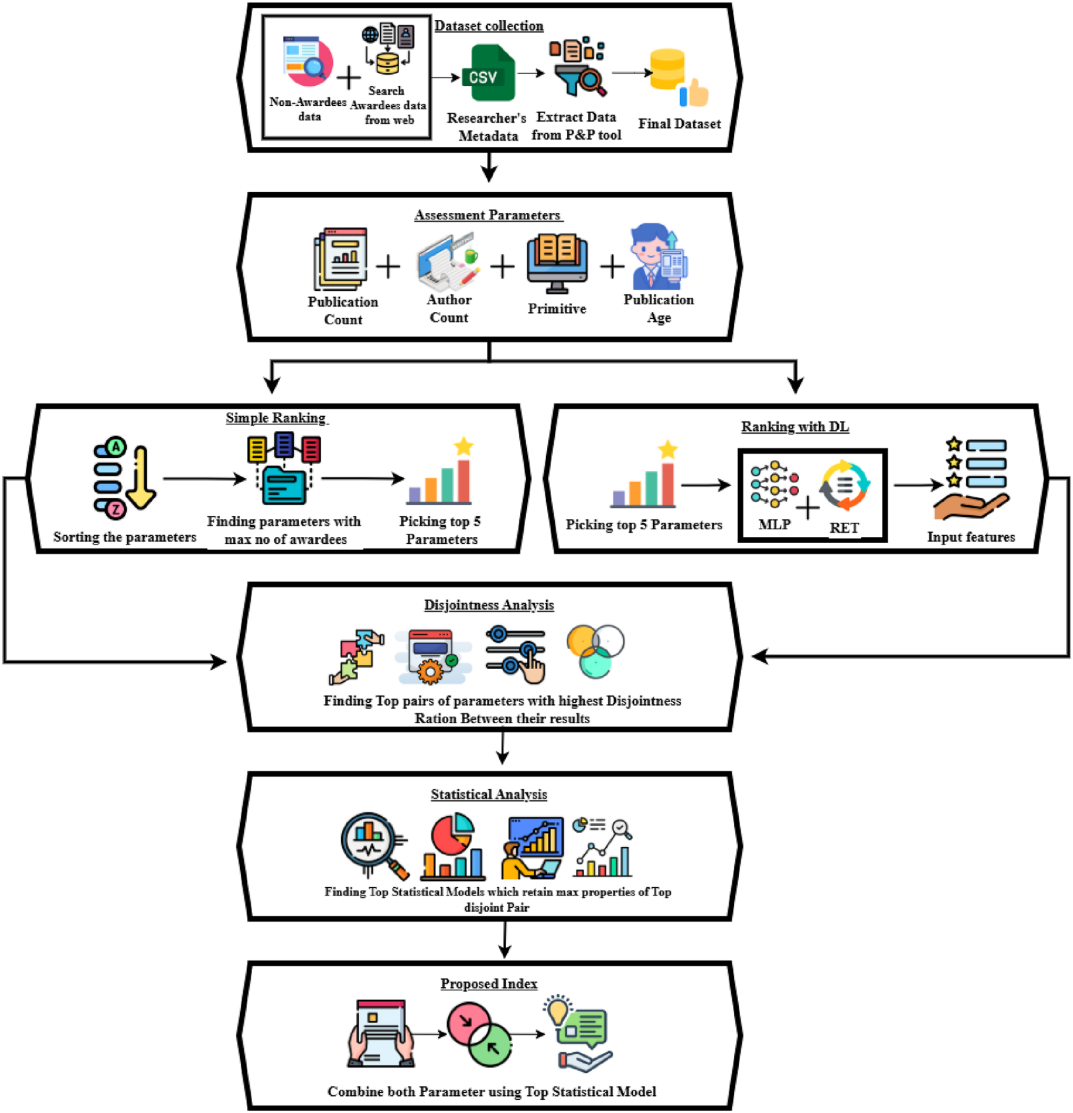
Architecture diagram of the proposed methodology. The figure illustrates the complete process, including dataset gathering, author assessment, ranking, disjointness analysis, and index formation.

Building on the findings from a comprehensive literature review, we now turn our attention to proposing a novel index for evaluating researchers in the Neuroscience domain. Figure 1 illustrates the architecture diagram, which outlines the sequential steps of our approach: (i) Dataset Collection, (ii) Parameter Calculation, (iii) Initial Ranking, (iv) Ranking through Deep Learning, (v) Disjointness Analysis, (vi) Statistical Analysis, and (vii) the Proposed Index. The following sections will offer a detailed explanation of each stage, highlighting its importance and role in the development of the new index.

### Dataset collection

For our experiments, we needed a dataset from a specific domain, and we chose Neuroscience due to its rich research history, significant contributions, and rapidly advancing field. Neuroscience, which focuses on diseases, disorders, and injuries involving the nervous system, is an area where scientists continually strive to prevent or treat issues affecting the brain and body. The vast amount of research in this field makes it an important area for ranking researchers. Its dynamic nature has also been the focus of prior studies^36^, making it an ideal subject for our work.

We compiled a dataset of 1060 records for this study, with an equal distribution of 530 awardees and 530 non-awardees. The awardee data was sourced from esteemed organizations such as the Society for Neuroscience (SFN), International Brain Research Organization (IBRO), Federation of European Neuroscience Societies (FENS), Cognitive Neuroscience Society (CNS), and Australasian Neuroscience Society (ANS), which honor researchers with various prestigious awards. The non-awardee data was adapted from previous datasets used by Ameer et al.^36^. A summary of the dataset statistics is provided in Table 1.

Data on awardees were gathered from the official websites of these societies, spanning over the last three decades, and included researchers’ names and award years. To obtain researcher data for the non-awardees, we used the “Publish or Perish” platform, which utilizes algorithms to extract metadata from Google Scholar. A “hold on” approach was applied to collect data for researchers prior to their award years. To ensure a balanced dataset, we matched the number of non-awardees for each year to the number of awardees. For example, if there were 15 awardees in 1999, we collected data on 15 non-awardees from before 1999.

Before performing any analysis, we conducted a thorough data cleaning process, especially for data extracted from platforms like Google Scholar, to remove inaccuracies and irrelevant information, often referred to as noise. This process involved verifying the data for accuracy and eliminating duplicate entries. To improve the dataset quality, two critical steps were taken. First, a filter was applied to ensure that all research articles were relevant to the Neuroscience field. Second, author disambiguation was conducted, addressing issues commonly associated with using Google Scholar data, as highlighted in previous studies^40^.

Our analysis involved two main samples: awardees and non-awardees. The full names of the 530 awardees were directly obtained from the society websites without requiring disambiguation. For the non-awardees, we used the dataset from Ameer et al.^36^. During the disambiguation process, we identified two primary cases: Case 1, where authors shared identical first and last names, necessitating verification; and Case 2, where authors had identical last names but different first names, requiring further evaluation. We followed established disambiguation methods from the literature^16,35^. In our dataset of 530 non-awardees, no cases involved identical first and last names, making Case 1 irrelevant.

During the disambiguation process, we encountered two primary challenges: (1) Identifying researchers with identical last names, which occurred in 23 instances, and (2) Differentiating authors who shared last names but had different first names. In this second category, we reviewed 50 cases and confirmed that 32 were distinct individuals. Additionally, we analyzed 41 cases of name variations and found that 25 were actually different representations of the same person. However, 23 instances involved identical last names, which required disambiguation. Our analysis showed that 32 out of 50 authors with the same last name but different first names were distinct individuals, while 25 out of 41 were variations of the same person. To maintain balance in the dataset, we added additional unique authors. The “Publish or Perish” tool was instrumental in verifying disambiguation, as it supported the inclusion of name variations and allowed for more accurate and comprehensive data collection.

**Table 1:** Dataset statistics.

Researchers metadata	Count
Total authors records	1060
Total awardees	530
Total non awardees	530
Total citation	25,855,493
Total publication	166,871

### Calculation of indices

In this section, we calculate 64 author assessment parameters, which are organized into four distinct categories based on their theoretical foundation and computational characteristics. These categories are: Primitive parameters, Publication and Citation count-based parameters, Author count-based parameters, and Publication age-based parameters.

The categorization was defined according to established scientometric principles and prior studies^41–44^. Specifically, Primitive parameters include basic quantitative measures such as total publications and total citations etc ; Publication and Citation count-based parameters incorporate combinations or ratios of publication and citation counts (e.g., H-index, G-index, $\hbox {H}_2$ index); Author count-based parameters adjust for collaboration effects by normalizing metrics based on the number of co-authors (e.g., fractional and pure H-indices); and Publication age-based parameters integrate temporal aspects of productivity, accounting for career length or publication recency (e.g., contemporary H-index, AR-index).

**Fig. 2:**
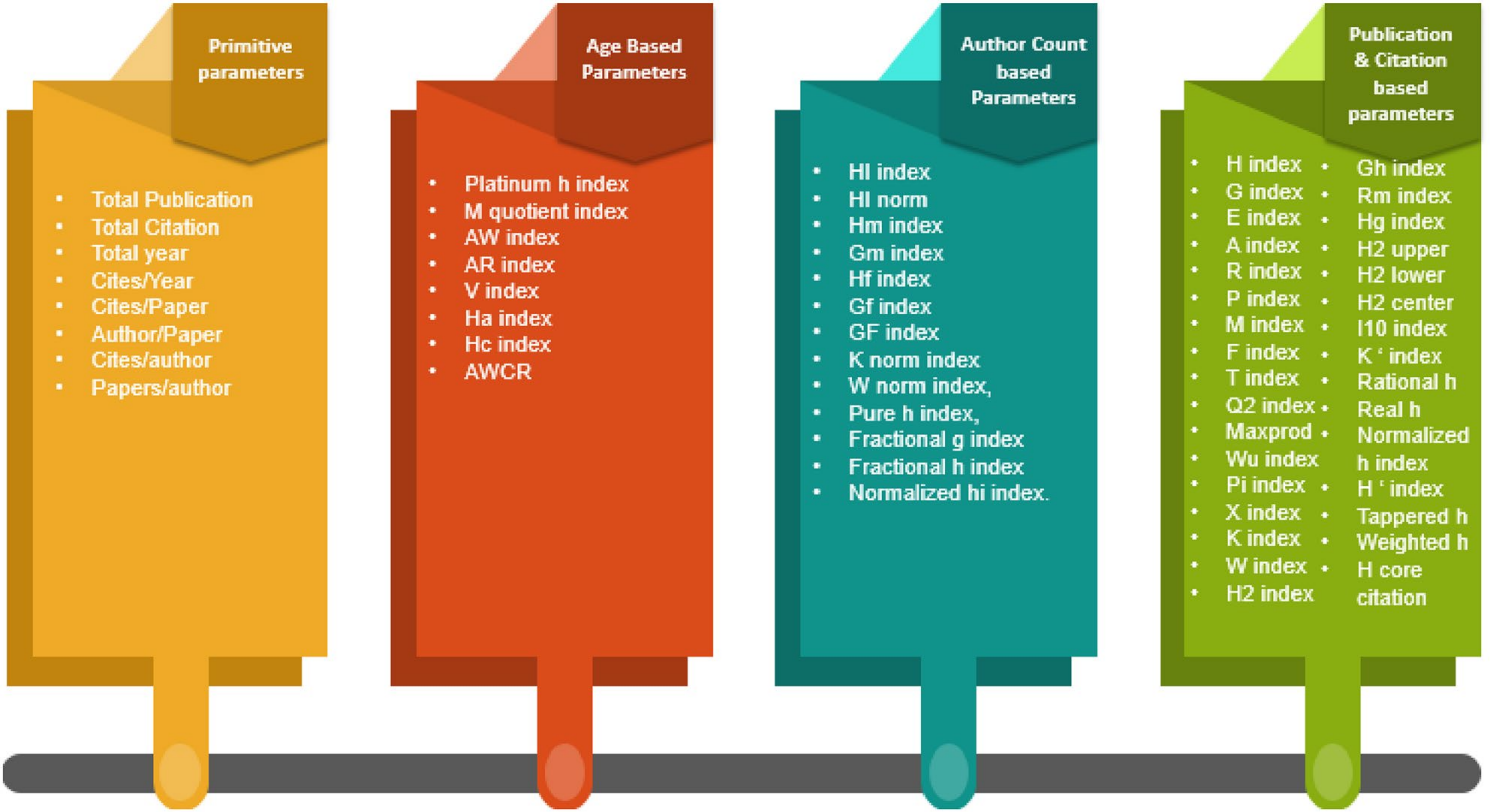
Types of parameters considered in this study, categorized into primitive, publication-count-based, author-count-based, and publication-age-based parameters.

A detailed overview of these categories, along with their corresponding indices, is provided in Fig. 2. To streamline the computation of these parameters, we developed a specialized application that extracts researcher data from Google Scholar profiles. This application computes all 64 parameters for each author using the data obtained from their profiles. The source code for this application is available at the following link: (https://github.com/ghulammustafacomsat/dataextraction).

### Simple ranking

In a simple ranking method, we start by creating individual author lists for each index, sorting them according to their respective index values. Once sorted, we determine how many awardees are included in the top 100 entries of each ranked list for every parameter. This process enables us to assess the effectiveness of each index in ranking authors. Ultimately, we select the top 5 parameters that identify the highest number of awardees within the top 100 records.

### Ranking with deep learning

Feature ranking is a crucial aspect of machine learning, contributing to tasks such as dimensionality reduction, improving model interpretability, reducing overfitting, optimizing predictions, and aiding feature selection. In this study, we utilize a multilayer perceptron (MLP) classifier combined with a modified recursive feature elimination technique to evaluate feature importance. The MLP is a neural network with multiple hidden layers, using ReLU activation functions in the hidden layers and softmax activation in the output layer. A loss function measures the difference between predicted and actual values, while backpropagation adjusts the model’s weights and biases to optimize performance. To prevent overfitting, batch normalization is applied after each hidden layer. The model consists of 10 hidden layers, each containing 10 neurons, and is trained using the Adam optimizer with a learning rate of 0.0003. Early stopping was employed to prevent overfitting by monitoring the validation loss during training. The categorical cross-entropy loss on the validation subset was tracked at each epoch, and training was automatically halted when no improvement in validation loss was observed for 10 consecutive epochs (patience = 10). The model state corresponding to the minimum validation loss was saved and used for subsequent evaluation to ensure optimal generalization. .

We also use a modified Recursive Elimination Technique (RET) to compute feature importance scores. RET is a well-established method for identifying the most relevant features that significantly influence a model’s performance^45,46^. The decision to combine MLP with RET is based on its simplicity and effectiveness in striking a balance between model complexity and performance, especially when working with smaller datasets. Deep learning models, if not properly regularized, can overfit smaller datasets due to their complexity^47^. RET helps mitigate this by reducing dimensionality, enhancing interpretability, and boosting generalization, while improving model efficiency. It systematically removes irrelevant or redundant features, ensuring that only the most impactful features remain.

The dataset was initially divided into training and testing sets in an 80:20 ratio. The training set was further split, allocating 80% for training and 20% for validation, ensuring a balanced class distribution across the subsets. The MLP classifier was trained on the training data, and predictions were made on both the validation and test sets, establishing the baseline accuracy with all features included.Subsequently, the feature removal process was carried out. One feature was excluded at a time, and the MLP classifier was retrained on the modified dataset. Predictions were made on both the validation and test sets, and the accuracy was recorded. The difference between this accuracy and the baseline accuracy was used as the importance score for the removed feature. This process was repeated for each feature over five epochs, and the importance score for each feature was calculated using the following equation:

1 $$\begin{aligned} F_{IS}=\frac{1}{5} \sum _{i+=20}^{100}(BLA_{i}-WOPA_{i}) \end{aligned}$$

where $F_{IS}$ represents feature important score, i represents the number of epochs, $BLA_{i}$ represents the baseline accuracy against the $i^{th}$ phase, and $WOPA_{i}$ represents the without-parameter accuracy of the $i^{th}$ phase.

After processing all the features, two lists are created: one for the feature names and the other for their corresponding importance scores. The features are then ranked according to these scores, and the top 5 features with the highest importance scores are selected.

### Disjointness

This study focuses on examining the disjointness among the results of top-performing indices. Disjointness is an important concept in scientometric and information retrieval studies, as it quantifies the independence between two ranked parameter sets and helps assess whether they capture complementary or redundant aspects of research performance^48–50^.

To investigate this, the top five parameters from each ranking method were selected, resulting in ten parameters overall. The disjointness ratio (DR) was then calculated for all possible pairs of these parameters to evaluate the degree of non-overlap in their top 100 ranked authors. The disjointness ratio between two parameters is defined as follows:

2 $$\begin{aligned} DR = 1 - \frac{|I1 \cap I2|}{|I1 \cup I2|} \end{aligned}$$

where $I1$ represents the first index in the pair, $I2$ the second, $|I1 \cap I2|$ denotes the number of overlapping authors in their top-100 lists, and $|I1 \cup I2|$ denotes the total unique authors identified by either parameter.

The disjointness ratio ranges from 0 to 1 (or equivalently 0% to 100%), where a higher value indicates greater independence between indices. For example, if the first parameter identifies 50 awardees within the top 100 records and the second parameter also identifies 50 awardees, with 25 authors common to both, the disjointness ratio would be 0.5 (50%). A value of 1 (100%) indicates no overlap, whereas a value of 0 denotes complete overlap between the results.

### Weight calculation of highest disjoint pair

Before proposing new indices based on statistical models, it is crucial to calculate the weight of each index. Simply inputting indices into the models assumes that all indices contribute equally to predicting awardees, but this assumption is flawed, as each index has a different impact on the prediction. To address this, we need to compute the weight of each top-performing index pair to reflect its significance in the model. For weight calculation, we have already ranked all indices using two methods, and an index’s rank in these rankings determines its weight. Specifically, an index’s position relative to the total number of indices (64 in this case) defines its weight. The following equations outline the procedure for calculating the weights of the parameters.

3 $$\begin{aligned} W_{s}=\frac{T_{i}-SR_{p}+1}{T_{i}} \end{aligned}$$

4 $$\begin{aligned} W_{d}=\frac{T_{i}-DR_{p}+1}{T_{i}} \end{aligned}$$

5 $$\begin{aligned} Pw = \frac{2 \times Ws \times Wd}{(Ws + Wd)} \end{aligned}$$

In the above equations, $T_{i}$ represent total no of indices, $SR_{P}$ represent position of parameter in simple Ranking, $DR_{p}$ represent position of parameter in Deep Learning Ranking, $W_{s}$ represent weight of parameter based on Simple Ranking, $W_{d}$ represent weight of parameter based on deep learning Ranking and $P_{w}$ represent final weight of parameter.

### Statistical analysis and proposed index

Through our disjointness analysis, we have identified and prioritized parameter pairs with the highest disjointness ratios. A high disjointness ratio between these pairs indicates a substantial difference in the awardee records they capture, suggesting that each parameter highlights a distinct set of features. This presents an opportunity to combine these unique feature sets, potentially leading to the creation of a new index that merges awardees from both sets, offering a more comprehensive view of the data. To merge the values of these pairs, we employed various statistical models, which are essential in data analysis across many research domains (Tzenios, 2023). In this study, we utilized advanced statistical techniques to extract meaningful insights from these pairs. By applying statistical analysis, we systematically explored the data, identified patterns, quantified relationships, and made well-informed inferences. Our objective was to integrate the top-performing pairs using several statistical methods, including the arithmetic mean, contra-harmonic mean, geometric mean, harmonic mean, Lehmer mean, logarithmic mean, root mean square, and trigonometric mean. These methods provided a holistic understanding of author rankings and their significance within the dataset. Detailed calculations for each method are presented in Table 2.

**Table 2:** Statistical methods and their acronyms.

Acronym	Method name	Formula
AM	Arithmetic mean	$AM = \frac{X_{1} + X_{2} + \dots + X_{n}}{n}$
HM	Harmonic mean	$HM = \frac{n}{\sum _{i=1}^{n} \frac{1}{X_{i}}}$
CHM	Contra–Harmonic mean	$CHM = \frac{X_{1}^{2} + X_{2}^{2} + \dots + X_{n}^{2}}{X_{1} + X_{2} + \dots + X_{n}}$
GM	Geometric mean	$GM = (X_{1} \times X_{2} \times \dots \times X_{n})^{\frac{1}{n}}$
LM	Logarithmic mean	$LM = \frac{1}{n} \times \left( \sum _{i=1}^{n} \log (X_{i}) \right)$
RMS	Root mean square	$RMS = \sqrt{\frac{X_{1}^{2} + X_{2}^{2} + \dots + X_{n}^{2}}{n}}$
TM	Trigonometric mean	$TM = \frac{\prod _{i=1}^{n} \sin (x_{i})}{\prod _{i=1}^{n} x_{i}}$

Furthermore, we employed the statistical methods presented in Table 2 to analyze the top-ranked pairs. By applying these methods, we computed the corresponding values for each pair, resulting in eight separate lists one for each statistical technique. We then compared these lists to identify the most impactful method for ranking the top pairs. This analysis also allowed us to determine the most suitable model for combining the features of the top-ranked pairs.

### Validation of proposed index

Various methods are employed in the literature to validate proposed indices. A common approach is to use data from renowned scientific academies, such as the National Academy of Sciences or the European Academy of Sciences, as benchmarks for assessing the effectiveness of these indices. Another popular method involves creating hypothetical scenarios where researchers simulate data for prominent professors from various institutions, allowing for the evaluation of the index’s performance in specific organizational contexts, such as universities and colleges. However, our analysis has identified an alternative validation method from the literature that provides a more realistic approach. This method involves evaluating the proposed indices based on prestigious awards received by individuals worldwide. These awards, spanning multiple fields and institutions, signify global recognition of excellence and competitiveness. While this approach offers a broader perspective, it does have certain limitations. By focusing solely on award recipients, it may overlook highly influential individuals who, despite their expertise, have not been formally recognized. This limitation highlights the need for a more comprehensive validation strategy that includes a diverse range of experts, regardless of award status. While this method provides a wider scope compared to institution-focused approaches, it still fails to capture the full breadth of expertise within a given field. Nevertheless, its focus on globally recognized achievements adds a valuable dimension to the evaluation process.

## Result and discussion

In this section, we present the experimental results obtained from the Neuroscience dataset.

### Simple ranking

**Fig. 3:**
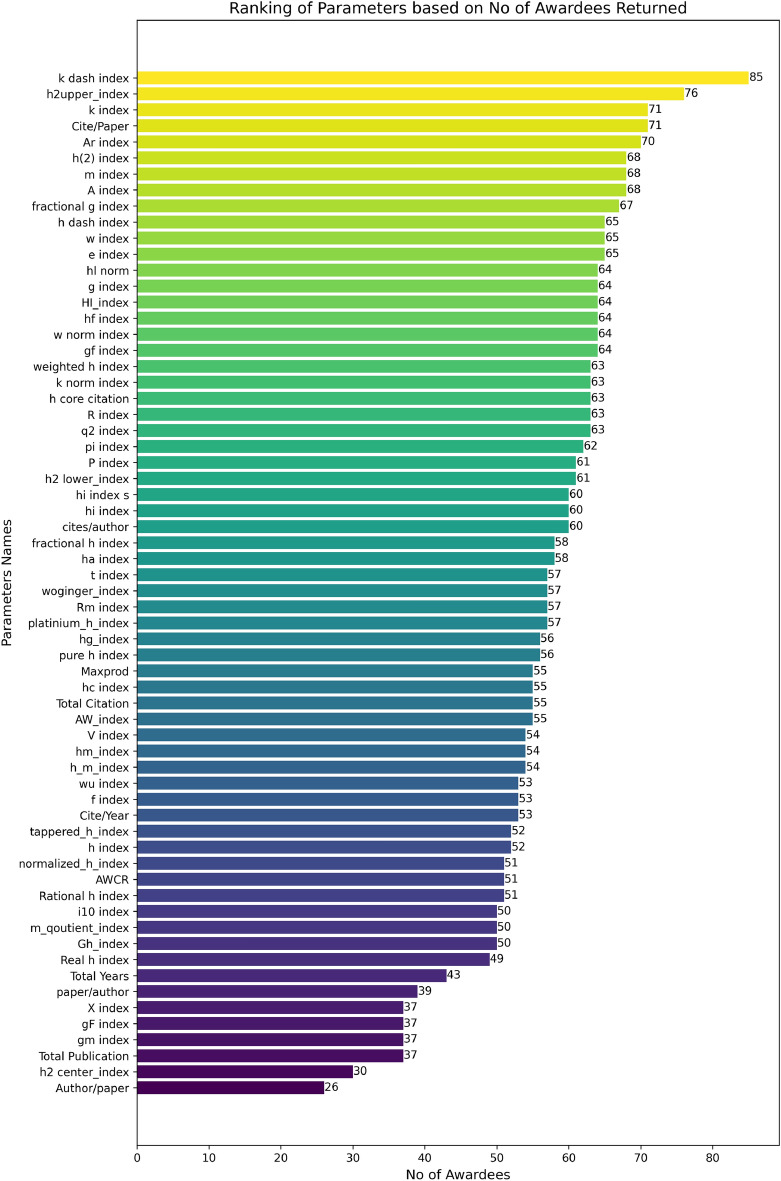
Simple ranking of parameters based on the number of returned awardees in the top 100 records. The colors represent a gradient where higher percentages are shown in yellow, transitioning through green and blue for lower scores. The color scale enhances visual clarity and does not indicate categorical distinctions.

We evaluated the influence of various indices on the ranking of awardees at the top of the list. Specifically, we analyzed the number of awardees appearing within the top 100 positions of the ranked list for each parameter. After gathering the relevant data, we assessed the rankings for each index individually and identified the positions of awardees within those rankings. Additionally, we determined the proportion of awardees included in the top 100 for each index. The results, depicted in Fig. 3, indicate that the h2 upper index, k dash index, Cite/Paper, Ar index, and h(2) index demonstrated the highest occurrences, ranging from 68 to 85%. Conversely, the h2 center index, gm index, and Author/Paper showed the weakest performance, with only 26–37% of awardees included.

### Ranking with deep learning

**Fig. 4:**
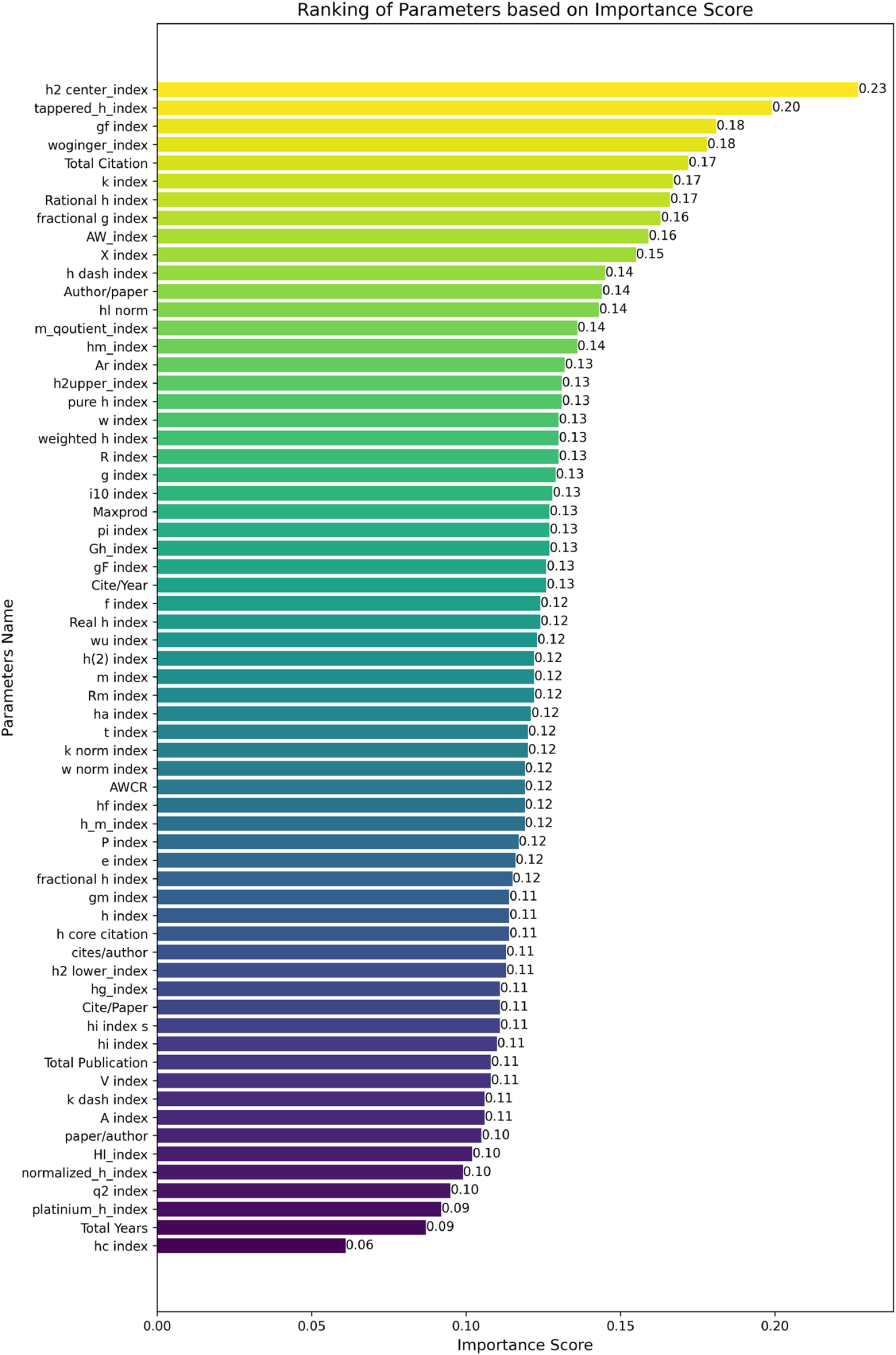
Ranking of parameters based on importance scores. The colors represent a gradient where higher importance scores are shown in yellow, transitioning through green and blue for lower scores. The color scale enhances visual clarity and does not indicate categorical distinctions.

In this section, we ranked the parameters based on their importance scores, calculated using the MLP classifier combined with the Modified Recursive Elimination Technique (as detailed in the “Methodology” section). A parameter’s importance score indicates its overall contribution to the model’s ability to classify awardees and non-awardees effectively. Figure 4 highlights a slightly different trend, with the top parameters h2 center index, tapered h index, gf index, woginger index, and Total Citation achieving high importance scores ranging from 0.172 to 0.227. These scores underscore their significant role in evaluating neuroscientific research. In contrast, parameters such as hc index, q2 index, platinum h index, and Total Years exhibited lower importance scores, ranging from 0.061 to 0.095, reflecting their reduced impact on model performance in this domain.

### Calculating disjointness ratio

In this section, we identified the highest-performing parameters and generated all possible pairwise combinations. For each pair, we created author ranking lists based on the two parameters and extracted the top 100 entries from these lists, recording the presence of awardees. We then calculated the disjointness ratio, which quantifies the proportion of unique awardees identified by each parameter pair. Table 3 reveals that the combination of the h2 upper index and k index achieved the highest disjointness ratio of 0.97, highlighting their distinct characteristics. In contrast, the combination of Total Citation and Ar index resulted in the lowest disjointness ratio of 0%, indicating a complete overlap in the awardees identified by these indices.

**Table 3:** Disjointness ratio on neuroscience dataset.

Pairs	DR	Pairs	DR
(h2upper_index, k index)	0.97	(tapered_h_index, h2upper_index)	0.64
(k dash index, gF index)	0.93	(tapered_h_index, h2 center index)	0.61
(h2 center index, k dash index)	0.94	(woginger_index, gF index)	0.58
(tapered_h_index, k dash index)	0.93	(h2 center index, gF index)	0.58
(k dash index, k index)	0.93	(h2upper_index, k dash index)	0.57
(Total Citation, k dash index)	0.92	(Total Citation, Cite/Paper)	0.57
(k dash index, Ar index)	0.92	(Cite/Paper, Ar index)	0.57
(h2upper_index, h2 center index)	0.82	(h2 center index, k index)	0.57
(Cite/Paper, h2 center index)	0.81	(tapered_h_index, woginger_index)	0.53
(woginger_index, k dash index)	0.79	(Total Citation, h2upper_index)	0.47
(Cite/Paper, k dash index)	0.77	(h2upper_index, Ar index)	0.47
(woginger_index, h2 center index)	0.77	(Cite/Paper, h2upper_index)	0.46
(Cite/Paper, k index)	0.77	(Cite/Paper, woginger_index)	0.42
(Total Citation, h2 center index)	0.76	(Total Citation, gF index)	0.37
(h2 center index, Ar index)	0.76	(Ar index, gF index)	0.37
(woginger_index, k index)	0.76	(Total Citation, woginger_index)	0.35
(tapered_h_index, k index)	0.73	(woginger_index, Ar index)	0.35
(Total Citation, k index)	0.72	(woginger_index, h2upper_index)	0.32
(Ar index, k index)	0.72	(Total Citation, tapered_h_index)	0.2
(gF index, k index)	0.72	(tapered_h_index, Ar index)	0.2
(Cite/Paper, gF index)	0.67	(tapered_h_index, gF index)	0.19
(Cite/Paper, tapered_h_index)	0.66	(Total Citation, Ar index)	0
(h2upper_index, gF index)	0.65		

### Weight calculation of highest disjoint pair

In the previous section, we identified the top-performing parameter pair: k index and h2 upper index. Before combining these parameters, it is crucial to calculate their respective weights using the formulas provided earlier. Based on these calculations, the weights for the top pair are 0.83 for the h2 upper index and 0.97 for the k index.

### Statistical analysis and proposed index

Ranking researchers effectively requires selecting the most distinct and complementary parameters to avoid redundant evaluations. In the realm of Neuroscience, the h2 upper index and the k index emerged as the most disjoint parameter pair, exhibiting a disjointness of 0.97. To determine the most effective method for integrating these two parameters, a detailed statistical evaluation was performed. Our analysis reveals that the Harmonic Mean (HM) model provides the most balanced and accurate rankings, achieving 80% accuracy for the top 20 and top 30 records, and maintaining a high accuracy of 78% for the top 100 records. The Harmonic Mean is particularly suited for combining indices with different impact levels, as it prevents one parameter from dominating the ranking while ensuring that both contribute meaningfully. Unlike the Arithmetic Mean, which gives equal weight to all values, the Harmonic Mean ensures that researchers with high impact in different dimensions (citation impact vs. sustained influence) are fairly represented. For example, consider two researchers:

- Researcher A has a strong h2 upper index, indicating a high number of highly cited publications.
- Researcher B has a strong k index, reflecting consistent contributions across multiple publications over time.

A single metric may undervalue one aspect while overvaluing another, leading to misleading rankings. The Hk index resolves this issue by integrating these aspects harmonically, ensuring that researchers with different strengths are equitably ranked. Table 4 compares various statistical models, illustrating the superiority of the Harmonic Mean, which achieves an average impact score of 76.8 significantly higher than alternatives like the Arithmetic Mean (61.5) and the Geometric Mean (63.5). Based on this analysis, we propose the Hk index, which harmonically integrates the h2 upper index and k index, ensuring that neither citation count nor sustained influence alone dominates the ranking process. A potential concern is the dependence on award-winning scientists for validation. While prestigious awards serve as strong indicators of research impact, we acknowledge that they do not capture all influential researchers. To address this, we compared Hk index rankings with both awardees and non-awardees, demonstrating that the index remains robust beyond formal recognitions. The final Hk index formula is given as:

6 $$\begin{aligned} HKindex = \frac{2 \times ((0.83 \times H2upperindex)(0.97 \times kindex))}{((0.83 \times H2upperindex) + (0.97 \times kindex))} \end{aligned}$$

By balancing citation-based influence and long-term research contribution, the Hk index offers a more refined and practical tool for ranking researchers, making it a valuable addition to the academic evaluation landscape.

**Table 4:** Result of statistical models on neuroscience dataset.

Models	TOP 10	TOP 20	TOP 30	TOP 40	TOP 50	TOP 60	TOP 70	TOP 80	TOP 90	TOP 100	Average impact
AM	40	55	63.3	57.5	62	61.6	67.1	70	67.7	71	61.5
HM	60	75	80	80	78	80	78.5	80	78.8	78	76.8
CHM	40	55	63.3	57.5	62	61.6	67.1	70	67.7	71	61.5
GM	40	60	66.6	65	64	66.6	67.1	67.5	67.7	71	63.5
LM	40	60	66.6	65	64	66.6	67.1	67.5	67.7	71	63.5
RMS	40	55	63.3	57.5	62	61.6	67.1	70	67.7	71	61.5
TM	20	25	40	47.5	50	51.6	52.8	56.25	54.4	55	45.2

### Conceptual explanation of $\hbox {H}_2$ upper, K, and HK indices

A deeper understanding of the selected indices and their integration is essential for interpreting the proposed HK index effectively. Each of the three indices-H2 upper, K index, and HK index captures a distinct yet complementary dimension of research performance.

**H2 upper index:** The H2 upper index is an advanced extension of the traditional h index that emphasizes the *depth of citation impact*. It identifies the largest number of papers (H2) that each have at least $\hbox {H}^2$ citations. This index highlights a researcher’s ability to produce multiple publications with exceptionally high citation counts, reflecting consistent, high-impact scholarship . It moves beyond simple productivity by rewarding depth rather than breadth of impact.

**K index:** The K-index evaluates scientific recognition through indirect influence within the scholarly network. Rather than counting direct citations, it measures how many highly cited authors have cited the researcher’s work. This approach captures prestige, influence, and intellectual reach within the academic community. A high K index indicates that an author’s contributions have shaped the work of other influential scientists, making it a strong indicator of scholarly recognition.

**Proposed HK-index:** The HK index harmonically integrates the H2 upper and K indices to create a more balanced and comprehensive evaluation metric. By combining the *intensity of citation impact* (H2 upper) with the *breadth of scholarly recognition* (K index), the HK index mitigates the limitations of relying on either dimension alone. The harmonic mean ensures that both parameters contribute proportionally, preventing dominance by one index and maintaining fairness across varying performance profiles. This balanced integration produces a more stable and interpretable ranking system that captures both sustained research excellence and academic influence across networks.

Collectively, these indices provide a multifaceted understanding of researcher performance, aligning quantitative impact with qualitative recognition to offer a holistic measure of academic achievement.

### Interpretability and practical utility of the Hk index

A critical factor in the adoption of any ranking metric is its interpretability and real-world applicability. While the Hk index is built upon statistical rigor, its practical implementation remains straightforward, making it a viable tool for various stakeholders in academia. Universities and academic institutions can use the Hk index for faculty promotions, tenure evaluations, and researcher recognition programs. Funding agencies can benefit from the Hk index’s ability to identify researchers with sustained, diverse, and high-impact contributions, rather than relying solely on citation-based rankings. Individual researchers can use the Hk index to gain insights into their relative standing in the academic community and assess the impact of their work beyond traditional indicators. One of the main concerns regarding new ranking indices is validation and usability. The Hk index was validated using a dataset of award-winning researchers, ensuring that it effectively highlights individuals recognized for their significant contributions. However, recognizing that awards do not always capture all influential researchers, the study also includes non-awardee comparisons to prevent bias. Additionally, a correlation analysis with traditional indices (such as the h index) was conducted to ensure that the Hk index remains consistent with established evaluation methods while offering a more nuanced perspective.

## Conclusion

This paper tackles the ongoing challenges in ranking scholars within the scientific community by critically evaluating a broad spectrum of parameters and proposing a novel index. Despite the availability of over 70 author-ranking parameters in the literature, encompassing both quantitative and qualitative dimensions, there remains no consensus on a definitive ranking metric. To address this issue, we adopt a holistic approach by analyzing 64 parameters using traditional ranking methods based on index calculations, complemented by advanced deep learning techniques. Rather than emphasizing a single top-performing parameter, we identify the five most effective parameters that consistently highlight awardees. Our experiments are conducted using a dataset from the Neuroscience domain. To refine the analysis further, we compute the disjointness ratio between the top-performing parameter and all possible combinations, selecting the two parameters with the highest disjointness. These top disjoint parameters are then analyzed using seven statistical models to identify the most effective method for their combination. Weight factors are incorporated into the index formula to reflect the relative importance of each parameter. The result of this study is the introduction of the Hk index, a new metric derived from the combination of the h2 upper index and the k index. The combination is supported by the Harmonic Mean, identified as the most effective statistical model. The Hk index provides an effective framework for evaluating scholarly impact within the Neuroscience domain. While it demonstrates strong performance based on our evaluation criteria, we acknowledge that its applicability may vary across different fields and require periodic recalibration over time. Additionally, the reliance on award-winning scientists as a validation standard introduces an inherent limitation scholarly impact is not solely determined by awards, and some highly influential researchers may not be recognized within our dataset. Future work could explore broader validation methods to mitigate this constraint. Nonetheless, this study has certain limitations. First, the proposed index is specifically tailored to the Neuroscience domain. As the field evolves, recalibration of weight factors and adjustments to the index may become necessary. Second, the validation process primarily focuses on award-winning researchers, potentially overlooking other influential contributors who have not received formal recognition.

### Future work

In future work, we plan to expand our research in two significant directions. First, we aim to include a broader range of published indices in our analysis to extend the scope and depth of the evaluation. Second, we intend to gather datasets from diverse disciplines, including Civil Engineering and Computer Science. This expanded dataset will allow for a comprehensive assessment of the existing 64 parameters and the newly proposed parameter across multiple fields. Additionally, to mitigate the dependency on award-winning researchers, we plan to explore alternative validation strategies. This includes incorporating datasets of highly cited researchers who have not received formal awards and leveraging expert opinions to identify influential scholars. By diversifying our validation benchmarks, we aim to ensure that the proposed ranking index is more generalizable and applicable across various academic domains.

## Data Availability

The dataset generated and/or analyzed during the current study is not publicly available due to privacy/ownership constraints but is available from the corresponding author on reasonable request. Please contact Dr. Ghulam Mustafa at ghulam.mustafa.ssc@stmu.edu.pk for data inquiries.
